# Quality of Life and Executive Function Deficits in Inflammatory Arthritis: A Comparative Study of Rheumatoid and Psoriatic Arthritis

**DOI:** 10.3390/healthcare13151928

**Published:** 2025-08-07

**Authors:** Cigdem Cekmece, Begum Capa Tayyare, Duygu Temiz Karadag, Selime Ilgin Sade, Ayse Cefle, Nigar Dursun

**Affiliations:** 1 Section of Occupational Therapy, Department of Therapy and Rehabilitation, Vocational School of Kocaeli Health Services, Kocaeli University, Umuttepe Campus, 41380 Izmit, Turkey; begum.capatayyare@kocaeli.edu.tr; 2 Division of Rheumatology, Department of Internal Medicine, Faculty of Medicine, Kocaeli University, 41380 Izmit, Turkey; duygu.temiz@kocaeli.edu.tr (D.K.); ayse.cefle@kocaeli.edu.tr (A.C.); 3 Department of Physical Medicine and Rehabilitation, Faculty of Medicine, Kocaeli University, 41380 Izmit, Turkey; ilgin.sade@kocaeli.edu.tr (S.I.S.); nigard@kocaeli.edu.tr (N.D.)

**Keywords:** rheumatoid arthritis, psoriatic arthritis, quality of life, activity daily living, executive functions

## Abstract

**Background/Objective:** Executive functions (EFs) are essential in the daily management of arthritis, as they influence treatment adherence, decision-making, and the ability to cope with disease-related challenges. The objective of this study was to compare EFs alongside functional status and quality of life in patients with rheumatoid arthritis (RA) and psoriatic arthritis (PsA) and examine their associations with disease activity and clinical variables. **Methods:** In this cross-sectional study, 140 patients (70 RA, 70 PsA) were assessed using the Stroop-TBAG, Wisconsin Card Sorting Test (WCST), and Adult Executive Functioning Inventory (ADEXI). Functional status and quality of life were measured with the Health Assessment Questionnaire (HAQ) and WHOQOL-BREF, respectively. Correlations with disease activity (DAS28-CRP), age, and disease duration were examined. **Results:** RA patients had significantly higher disease activity and longer disease duration. They showed poorer performance on the Stroop Test (color–word time: 61.6 ± 14.8 vs. 52.4 ± 10.9 s, *p* < 0.001; errors: 3.2 ± 2.1 vs. 2.1 ± 1.5, *p* = 0.001), more WCST perseverative errors (*p* = 0.002), and higher ADEXI inhibition scores (13.9 ± 2.5 vs. 12.9 ± 3.0, *p* = 0.013). DAS28-CRP was correlated with EF impairments, disability, and poorer quality of life in RA (*p* < 0.05). In PsA, EFs remained relatively stable, although higher disease activity was associated with worse HAQ scores (*p* = 0.001). Treatment type was not linked to EF, but patients on combination therapy reported lower physical (*p* = 0.009) and psychological (*p* = 0.014) quality of life, along with higher HAQ scores (*p* = 0.016). **Conclusions:** This study revealed that patients with RA exhibit more pronounced executive dysfunction, along with lower ADL skills and quality of life compared to those with PsA. These findings highlight the need for multidimensional assessment strategies in inflammatory arthritis, especially in RA, where cognitive and functional outcomes are closely tied to clinical burden.

## 1. Introduction

Rheumatoid arthritis (RA) is a chronic autoimmune disease that primarily targets connective tissue and synovial joints. Although RA is recognized globally, its reported prevalence varies considerably across populations, ranging from 0.24% to 2% [1]. The disease exhibits a higher incidence in women and typically presents around the fifth decade of life. In addition to articular involvement, RA is associated with systemic manifestations that contribute to increased morbidity [2]. Psoriatic arthritis (PsA) is a heterogeneous inflammatory arthropathy associated with psoriasis. While the pooled prevalence of PsA among individuals with psoriasis is approximately 19.7%, some studies have reported rates as high as 41.8%, underscoring significant variability among populations [3]. PsA typically manifests between the ages of 40 and 50, affecting both sexes equally [4]. Similar to RA, PsA extends beyond joint involvement and may affect extra-articular systems such as the eyes and gastrointestinal tract. Additionally, PsA is associated with several comorbidities, including cardiovascular disease, non-alcoholic fatty liver disease, and metabolic syndrome [5]. Chronic inflammation, along with the long-term effects of both the disease and its treatment, predisposes individuals with RA and PsA to an increased risk of comorbid conditions and contributes to a reduction in life expectancy by several years [6].

One of the primary goals in managing chronic inflammatory diseases is to alleviate patients’ complaints and enhance their quality of life. Clinical observations indicate that patients with RA frequently experience significant difficulties in performing activities of daily living. Persistent pain due to synovitis and functional impairment resulting from progressive joint destruction are among the key factors that negatively impact the quality of life in this population. Functional limitations, reduced physical capacity, and disease-related fatigue can also make treatment adherence difficult and compromise self-care abilities [7]. These challenges not only diminish overall well-being but are also associated with cognitive impairments. Similarly, in PsA, disease progression may result in joint inflammation and structural damage, leading to varying degrees of physical disability. Studies have shown that functional deterioration in PsA can be comparable to that observed in RA, with both conditions significantly disrupting patients’ ability to carry out daily activities and maintain independence [8].

Emerging evidence suggests that, beyond musculoskeletal involvement, RA and PsA may also negatively impact cognitive and executive functions (EFs). Chronic systemic inflammation, ongoing pain, and psychosocial stressors are believed to contribute to cognitive deficits in these populations—particularly in areas such as attention, memory, and executive control [9,10,11]. While cognitive dysfunction has been more extensively studied in RA, recent data increasingly highlight similar impairments in PsA, although they remain underrecognized [10,12]. Despite these observations, few studies have directly compared EF profiles between RA and PsA patients using standardized neuropsychological tests and validated self-report tools.

Executive functions are multifaceted and typically assessed through a combination of neuropsychological instruments that capture key domains, including working memory, cognitive flexibility, and inhibitory control. Diamond’s model [13], currently the most widely endorsed framework for conceptualizing EFs [14], posits three core components: (i) inhibition, defined as the ability to regulate attention and behavior by resisting impulsive responses [15]; (ii) working memory, the capacity to hold and manipulate information over brief periods [16]; and (iii) cognitive flexibility, the ability to shift perspectives, alternate between tasks, and adapt strategies in response to changing conditions [17]. Although functionally distinct, these processes typically operate interactively to facilitate goal-directed behavior [13,15].

The present study aimed to comprehensively assess EF in patients with RA and PsA using the Wisconsin Card Sorting Test (WCST), the Stroop Test (TBAG form), and the Adult Executive Function Inventory (ADEXI). Additionally, functional status and quality of life are evaluated through the Health Assessment Questionnaire (HAQ) and the World Health Organization Quality of Life Instrument—Short Form (WHOQOL-BREF). By evaluating these domains collectively, this study sought to elucidate disease-specific cognitive–affective profiles and their functional correlates.

## 2. Methods

### 2.1. Participants

We conducted a cross-sectional observational study involving patients with RA and PsA from the outpatient clinic of the Department of Rheumatology at Kocaeli University Hospital. Patients with RA, who met the 2010 classification criteria of the American College of Rheumatology (ACR)/European Alliance of Associations for Rheumatology (EULAR) [18], and patients with PsA, who met the 2006 Classification Criteria for Psoriatic Arthritis (CASPAR) [19], were consecutively recruited between January 2025 and May 2025. To ensure greater comparability with RA in terms of joint involvement and treatment characteristics, PsA patients with joint patterns specific to PsA—such as axial disease, distal interphalangeal joint involvement, or arthritis mutilans—were excluded from the study.

Comparability between patients was ensured with respect to treatment type (biological and non-biological), age, sex, and educational status. Demographic data (sex, age, occupation, years of education, and marital status), clinical information (comorbidities, current medications, disease duration, and Disease Activity Score 28 using C-reactive protein (DAS28-CRP)), and laboratory findings were extracted from patients’ medical records. Physical examinations and disease activity assessments were performed by a single rheumatologist who was not involved in the cognitive evaluations, thereby minimizing potential bias. Patients were excluded as follows: if they had rheumatic diseases other than RA or PsA; if they had any chronic neurological or systemic condition (e.g., stroke, chronic heart failure, dementia, epilepsy); if they were taking medications known to affect cognitive function (e.g., antidepressants); or if they had perceptual or communication impairments, including color blindness.

All questionnaires were self-administered by the participants with assistance provided by a researcher and an experienced physiotherapist when needed. Evaluations were conducted in a quiet, temperature-controlled room, free from external distractions. The total duration for administering the tests and questionnaires was approximately 45 min per patient. To ensure consistency and standardization, all neuropsychological assessments were conducted by the same researcher, who holds formal certification in test administration and was blinded to the participants’ clinical diagnosis (RA or PsA) to minimize potential assessment bias.

An a priori power analysis was conducted using G*Power software (version 3.1.9.2; Kiel University, Kiel, Germany) to determine the required sample size for detecting differences in executive function (EF) abilities between patients with RA and those with PsA. The analysis was based on a two-tailed independent samples t-test, assuming a medium effect size (Cohen’s d = 0.5), an alpha level of 0.05, and a statistical power (1–β) of 0.90. The minimum required sample size was calculated to be 70 participants per group. To account for potential dropouts, the target enrollment was set at 75 participants in each group.

All participants were fully informed about the study procedures, and written informed consent was obtained prior to participation. The study protocol was approved by the Ethics Committee of Kocaeli University (approval number: KU GOKAEK-2025/02/09).

### 2.2. Assessment Tools

EF abilities of all patients were assessed using the WCST, the Stroop-TBAG Test, and the ADEXI. Activities of daily living (ADL) were evaluated with the HAQ, while quality of life was assessed using the WHOQOL-BREF.

Executive function (EF) abilities were assessed using the WCST, the Stroop-TBAG, and the ADEXI. Activities of daily living (ADL) were evaluated using the HAQ, and quality of life was measured with the WHOQOL-BREF instrument.

#### 2.2.1. The Wisconsin Card Sorting Test (WCST)

The Wisconsin Card Sorting Test (WCST) is a widely used neuropsychological instrument for the assessment of EFs [20]. It is particularly valuable for evaluating cognitive flexibility, abstraction, and reasoning abilities [21]. One of its primary strengths is its sensitivity to detecting and characterizing executive dysfunction, especially in clinical populations [22]. The Turkish adaptation and validation of the WCST were performed by Karakas et al. [23]. The test consists of two decks of response cards (each containing 64 cards) and four stimulus cards, yielding a total of 128 response cards. Each card displays figures that vary in three attributes: color (red, green, yellow, or blue), number (one to four), and shape (cross, circle, star, or triangle). The arrangement, orientation, and positioning of the stimulus and response cards are standardized to ensure consistency across administrations.

#### 2.2.2. The Stroop-TBAG Test

The Stroop Test is a widely used neuropsychological instrument for evaluating EFs, particularly in the ability to adapt perceptual processing to changing task demands under conditions of interference, in inhibiting automatic responses in favor of goal-directed actions, and in maintaining sustained attention [24]. Although several versions of the Stroop Test exist, they are all based on the same core principle: participants are asked to verbally identify the ink color of color words that are printed in an incongruent color (e.g., the word “red” printed in blue ink). The Stroop-TBAG version, adapted and validated for the Turkish population by Karakaş et al. in 1999 [25], integrates elements from both the original Stroop and the Victoria versions. It consists of five sequential tasks administered under timed conditions. In the first task, participants are required to read color names printed in black ink (hereafter referred to as “color words”). In the second task, they are asked to read color names printed in colored ink. In the third task, participants must name the colors of circles painted in four different colors (yellow, blue, red, green). The fourth task requires participants to name the ink color of words unrelated to color names but printed in different colors (for example, the word “weak” printed in blue ink). In the fifth and final task, referred to as the “color–word” condition, participants are instructed not to read the color words printed in an incongruent color (e.g., the word “yellow” printed in blue ink) but instead to name the ink color. In other words, participants must perform a less automatic task (naming the ink color) while inhibiting the more automatic tendency to read the word itself [24,26,27]. Performance in each condition is evaluated based on completion time. Although the Stroop Test is applicable across various age groups, fluent reading skills are required for its administration.

#### 2.2.3. The Adult Executive Functioning Inventory (ADEXI)

The ADEXI was developed by Holst and Thorell in 2018 to assess core executive functions, specifically working memory and inhibition [28]. Unlike broader executive functioning assessments, ADEXI provides a focused and precise evaluation by directly targeting these fundamental components. Its brevity and ease of administration make it particularly useful for both general and clinical populations, especially for individuals who may have difficulty completing longer assessment protocols [29]. The Turkish adaptation and validation of the ADEXI were conducted by Alpay and Kızıloz [30].

#### 2.2.4. Health Assessment Questionnaire (HAQ)

HAQ was modified initially by Pincus and colleagues [31], and its validity and reliability for the Turkish population were established by Kuçukdeveci et al. [32]. HAQ is designed to evaluate an individual’s functional status and ability to perform ADL. It is beneficial for assessing physical functioning in patients with chronic conditions such as rheumatic diseases. The questionnaire covers eight domains: eating, dressing, hygiene, gripping, walking, reaching, bathing, and household activities. It also evaluates the degree of assistance required. Each item is scored on a 4-point scale ranging from 0 (no difficulty) to 3 (unable to do). HAQ is a well-validated and widely used instrument in both clinical research and routine patient monitoring.

#### 2.2.5. World Health Organization Quality of Life Instrument—Short Form (WHOQOL-BREF)

The WHOQOL-BREF is a 26-item scale that assesses individuals’ quality of life across four primary domains: physical health, psychological health, social relationships, and environmental factors [33]. Designed to be culturally adaptable, the WHOQOL-BREF offers a practical and standardized method for evaluating overall well-being and quality of life. The Turkish adaptation and validation of the scale were conducted by Eser et al. [34]. WHOQOL-BREF is widely used in clinical practice, public health research, and individual health assessments.

### 2.3. Statistical Analysis

Descriptive statistics for clinical and demographic characteristics were summarized as frequencies and percentages for categorical variables and as mean ± standard deviation (SD) or median (interquartile range—IQR) for continuous variables, depending on data distribution. The normality of numerical data was evaluated using visual inspection and the Shapiro–Wilk test. Differences in demographic, clinical, and laboratory data between RA and PsA patients were analyzed using the Mann–Whitney U test or independent samples t-test for continuous variables and the chi-square test for categorical variables. Based on DAS28-CRP scores, RA and PsA patients were categorized into four disease activity groups: remission (≤2.6), low disease activity (>2.6–≤3.2), moderate disease activity (>3.2–≤5.1), and high disease activity (>5.1). To compare assessment tool results among these groups, one-way ANOVA was used for continuous variables with a normal distribution, while the Kruskal–Wallis H test was applied for non-normally distributed variables. Categorical variables were analyzed using Pearson’s chi-square test. Similar analyses were also conducted based on treatment groups: conventional synthetic DMARDs (csDMARDs), biologic DMARDs (bDMARDs), and a combination of both. For correlation analysis, Spearman or Pearson correlation analyses were conducted to examine relationships between assessment tool results with age, disease duration, and DAS28-CRP in RA and PsA Patients.

Participants with incomplete questionnaire or cognitive test data were excluded from the final analysis, and only complete datasets were included. All statistical analyses were conducted using SPSS version 20.0 (IBM Inc., Chicago, IL, USA). Statistical significance was defined as a two-sided *p*-value < 0.05.

## 3. Results

**Figure 1:**
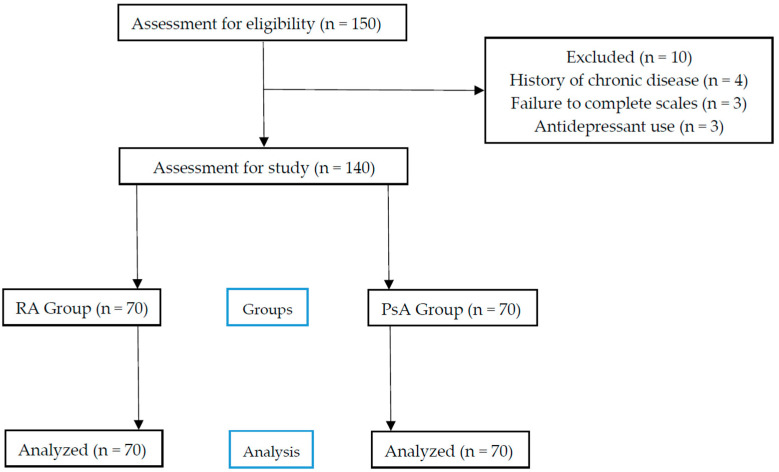
Flow chart.

A total of 150 patients diagnosed with either RA or PsA, aged between 18 and 70 years, were initially considered for inclusion. Of these, 10 patients were excluded due to chronic illness (*n* = 4), early withdrawal before completing assessments (*n* = 3), or the use of antidepressants (*n* = 3). The final sample consisted of 140 participants, equally divided into RA (*n* = 70) and PsA (*n* = 70) groups (Figure 1).

### 3.1. Sociodemographic and Clinical Characteristics

As shown in Table 1, no statistically significant difference was found between the groups in gender distribution (*p* = 0.065), although females predominated in both groups. Employment status differed significantly between groups (*p* = 0.011), with a higher proportion of employed patients in the PsA group and a higher proportion of homemakers in the RA group. No group differences were found in marital status (*p* = 0.309), comorbidity (*p* = 0.307), or medication type (*p* = 0.085). While age and years of education were similar across groups, disease duration was significantly longer in the RA group (*p* = 0.006), and DAS28-CRP scores were higher in RA patients (*p* = 0.036). Among laboratory parameters, only platelet count was significantly higher in RA patients (*p* = 0.031). Although the overall distribution of medication types did not differ significantly between the groups (*p* = 0.085), conventional synthetic DMARDs (csDMARDs) were more frequently used in the RA group (58.6%) compared to the PsA group (50.0%). In contrast, the use of biological agents was more common among PsA patients (14.3%) than among those with RA (7.1%).

### 3.2. Cognitive and Functional Assessments

Table 2 summarizes neuropsychological and functional assessments. RA patients performed significantly worse on the Stroop-TBAG Test across all subcomponents, including color–word time, errors, and self-corrections (all *p* < 0.01). WCST scores were also significantly poorer in the RA group in nearly all domains (*p* < 0.05), except for non-perseverative errors (*p* = 0.131) and failure to maintain set (*p* = 0.794). ADEXI scores showed significantly higher inhibition difficulty in RA compared to PsA (13.9 ± 2.5 vs. 12.9 ± 3.0, *p* = 0.013), with no significant difference in working memory (*p* = 0.166). PsA patients reported significantly better physical health scores on the WHOQOL-BREF (*p* = 0.041), while other subdomains showed no group differences (*p* > 0.05 for all). HAQ scores were higher in the RA group, though not statistically significant (10.7 ± 10.2 vs. 8.2 ± 9.0, *p* = 0.165).

### 3.3. Associations Between Clinical and Cognitive Variables

As presented in Table 3, age was significantly associated with poorer cognitive performance in both groups, with a more consistent pattern observed among RA patients. In RA, age positively correlated with Stroop color–word time and several WCST error parameters, including total responses, total errors, perseverative responses and errors (r values ranging from 0.383 to 0.498, all *p* ≤ 0.001). Conversely, age showed negative correlations with WCST accuracy and conceptual reasoning indicators, such as total correct responses, number of categories completed, and conceptual-level responses (r = −0.281 to −0.325, *p* < 0.05). Similar but weaker associations were noted in the PsA group.

Disease duration was positively associated with WCST perseverative errors in RA (r ≈ 0.27, *p* < 0.05), and with Stroop color–word time in PsA (r = 0.347, *p* = 0.003), suggesting that longer disease duration may contribute to deficits in processing speed and cognitive flexibility.

In RA, higher DAS28-CRP scores were linked to more Stroop errors, greater WCST perseverative errors, and poorer physical health on the WHOQOL-BREF (r values around 0.275 to 0.501, all *p* < 0.05). Additionally, higher DAS28 scores were associated with elevated ADEXI inhibition scores, indicating impaired executive control. In PsA, DAS28 was significantly correlated with increased HAQ scores (r = 0.521, *p* < 0.001), reflecting reduced physical functioning.

### 3.4. Cognitive Function Based on Disease Activity

RA and PsA patients were stratified into remission, low, and moderate disease activity groups based on DAS28-CRP scores. Only one RA patient fell into the high dis-ease activity category; therefore, this case was excluded from further group comparisons to maintain consistency across the cohorts. Cognitive test outcomes stratified by DAS28-CRP subgroups are presented in Table 4 and Table 5. Among patients with RA, Stroop and WCST performance did not significantly differ across disease activity groups. However, ADEXI inhibition scores were significantly worse in the moderate activity group compared to the others (*p* = 0.043). Post hoc Bonferroni analysis revealed that patients in remission had significantly better inhibition scores than those with moderate disease activity (mean difference = 1.9, 95% CI: 0.33 to 3.4, *p* = 0.012). In addition, WHOQOL-BREF physical and psychological health scores declined significantly with increasing disease activity (*p* = 0.002 and *p* = 0.020, respectively). Specifically, remission patients scored significantly higher than those with moderate activity in both domains (physical health—MD = 5.6, 95% CI: 2 to 9.3, *p* < 0.001; psychological health—MD = 2.8, 95% CI: 0.15 to 5.5, *p* = 0.035). In contrast, HAQ scores increased markedly (*p* < 0.001), indicating worsening physical function and quality of life. Post hoc comparisons confirmed a significantly lower HAQ score in the remission group compared to the moderate group (MD = 8.5, 95% CI: 2.4 to 14.6, *p* = 0.003).

In the PsA cohort, no significant differences were observed across disease activity groups in Stroop, WCST, or ADEXI performance. Similarly, WHOQOL-BREF domain scores did not vary significantly. However, HAQ scores showed a significant increase with higher disease activity (*p* = 0.001), reflecting greater disability. Post hoc analysis indicated that patients in remission had significantly lower HAQ scores compared to those with moderate activity (MD = 11.0, 95% CI: 5.2 to 17.0, *p* < 0.001).

### 3.5. Cognitive Function Based on Treatment Groups

As shown in Table 6, patients were also categorized according to treatment regimen. No statistically significant differences were observed in cognitive test scores (Stroop, WCST, ADEXI) across treatment groups. However, significant differences emerged in quality-of-life measures: patients receiving combination therapy had lower scores in the WHOQOL-BREF physical (*p* = 0.009) and psychological (*p* = 0.014) domains. Post hoc Bonferroni comparisons indicated that patients receiving combination therapy had significantly lower scores in the WHOQOL-BREF physical health domain compared to both the csDMARD group (mean difference = 3.5, 95% CI: 0.8 to 6.3, *p* = 0.007) and the biologic DMARD group (mean difference = 4.9, 95% CI: 0.7 to 9.1, *p* = 0.017). Similarly, psychological health scores were significantly lower in the combination therapy group compared to csDMARD (mean difference = 2.3, 95% CI: 0.4 to 4.3, *p* = 0.014) and biologic DMARD groups (mean difference = 3.8, 95% CI: 0.8 to 6.8, *p* = 0.008). Additionally, HAQ scores were significantly higher in this group (*p* = 0.016), indicating greater functional disability.

Post hoc Bonferroni comparisons indicated that this difference was driven by significantly higher HAQ scores in the combination therapy group compared to the csDMARD group (mean difference = −7.7, 95% CI: −12.0 to −3.4, *p* < 0.001).

## 4. Discussion

This study aimed to compare EFs in patients with RA and PsA using validated neuropsychological and self-report instruments and to explore how these cognitive outcomes relate to disease activity, functional status, and quality of life. To our knowledge, this is one of the few studies to directly assess and contrast EF domains in RA and PsA populations, with a specific focus on inhibition, cognitive flexibility, and working memory. Our findings revealed that EF impairments were more pronounced in RA patients compared to those with PsA and that these deficits were closely associated with disease activity, functional limitations, and perceived quality of life.

RA patients showed significantly poorer performance than PsA patients across all Stroop and most WCST metrics, indicating greater difficulty in inhibition, cognitive flexibility, and concept formation. These findings are consistent with previous studies reporting higher rates of executive dysfunction in RA, particularly among older individuals and those with elevated disease activity [9,35]. Notably, our data revealed strong correlations between EF impairments and clinical variables such as age, disease duration, and DAS28-CRP scores. When patients were stratified by disease activity, those with moderate RA activity showed worse inhibition (as measured by ADEXI), along with lower physical and quality-of-life scores. These results echo those of McDowell et al., who demonstrated that even moderate inflammation can negatively impact cognitive control, possibly through neuroinflammatory mechanisms involving cytokines such as TNF-α and IL-6 [35]. In contrast, PsA patients exhibited relatively preserved EF, which aligns with prior research suggesting that cognitive involvement in PsA is generally milder and more selective—often limited to attention or processing speed [36,37]. The observed disparity may reflect distinct neuroimmune profiles between the two diseases, with RA more prone to chronic systemic inflammation that may affect central executive networks.

In this study’s multidimensional evaluation, we observed that increasing disease activity was associated with greater impairment in ADL and quality of life, especially in RA. While HAQ scores worsened with disease activity in both groups, only RA patients demonstrated significant declines in the physical and psychological domains of the WHOQOL-BREF. These findings suggest that disease activity in RA has a broader impact, affecting not only physical functionality but also emotional well-being and cognitive control. This is supported by earlier studies indicating that individuals with higher DAS28 scores are more likely to experience severe disability and lower quality of life [38,39,40,41,42,43,44]. Consistent with our findings, Omma et al. and Ahmed & Amen reported strong associations between disease activity and functional disability in RA, as assessed by HAQ. Similarly, Husted et al. [45] showed reduced quality of life in both RA and PsA, though the effect was more substantial in RA. Our data extend these findings by demonstrating that cognitive impairments in RA may act as a mediator linking disease severity to diminished well-being.

Despite differences in treatment regimens, we did not observe significant variation in EF outcomes across csDMARD, biologic, or combination therapy groups. However, patients receiving combination therapy reported significantly worse physical and psychological quality of life, along with higher HAQ scores. These findings likely reflect greater underlying disease severity rather than a direct cognitive effect of treatment. This interpretation aligns with the RESIST trial and reviews by Fazel et al. and Sharma & Chen, which found limited evidence for direct neuroprotective effects of DMARDs, including TNF inhibitors [46,47]. Nonetheless, emerging data suggest that TNF-α blockers may exert central effects beyond their peripheral anti-inflammatory properties. Evidence suggests that RA patients treated with anti-TNF agents may have a reduced risk of developing Alzheimer’s disease compared to controls, whereas such an association has not been observed with other DMARDs [48]. Moreover, an fMRI study demonstrated that anti-TNF therapy led to significant reductions in brain activation within three days—preceding any measurable improvement in joint symptoms—suggesting a possible direct CNS effect [49] The primary focus of our study was to compare EF profiles independent of pharmacological effects. Therefore, in the study design, we carefully ensured that the RA and PsA groups were comparable in terms of both csDMARD and bDMARD exposure. Although our findings did not reveal EF differences attributable to treatment class, these contrasting observations highlight the need for future mechanistic studies to clarify the neurocognitive impact of anti-inflammatory therapies in RA.

Proinflammatory cytokines such as IL-1β, IL-6, and TNF-α have been implicated in cognitive dysfunction in RA by affecting neural circuits, including those in the prefrontal cortex. Elevated IL-1β levels in the cerebrospinal fluid of RA patients suggest intrathecal immune activation [50] These cytokines can impair synaptic plasticity and modulate glutamatergic and GABAergic transmission, leading to deficits in executive functions such as working memory and attention [51,52,53]. IL-6 overexpression has also been linked to age-related cognitive decline and synaptic loss [54]. Clinically, higher peripheral levels of IL-6 and TNF-α have been associated with poorer cognitive performance in RA patients with active disease [55]. However, as our study population consisted mainly of patients with low disease activity and minimal systemic inflammation, these mechanisms may not have played a major role in our findings. We include this discussion to provide biological context and emphasize the importance of future studies in patients with higher inflammatory burden.

This lack of significant differences could be due to the relatively small sample size in each treatment subgroup or the possibility that cognitive changes occur independently of pharmacological modulation. Notably, in some subgroup analyses—particularly within the PsA cohort—no significant associations were found between EF performance and quality of life or HAQ scores. One possible explanation is that the relatively mild EF impairment in PsA may not be severe enough to impact daily function in a measurable way. Alternatively, different compensatory mechanisms or psychological resilience may buffer against cognitive impact in this population.

The observed cognitive, functional, and quality-of-life impairments in RA—especially in those with higher disease activity—underscore the need to integrate cognitive screening and psychosocial assessments into routine care. Executive dysfunction can impair treatment adherence, reduce self-management capacity, and exacerbate disability. Multidisciplinary management strategies, that address not only physical symptoms but also cognitive and psychological domains, may lead to improved outcomes. Future studies should include longitudinal designs and biomarker analyses to clarify the temporal and mechanistic relationships between systemic inflammation, neurocognitive impairment, and treatment response. Incorporating healthy control groups and more diverse PsA subtypes could also enhance the generalizability of results.

This study has several limitations. First, RA patients had longer disease duration and higher disease activity, which may have independently influenced cognitive outcomes. Second, the absence of a healthy control group limits our ability to determine whether observed impairments exceed those expected with aging or comorbidities. Lastly, although we excluded PsA phenotypes with axial or distal joint involvement for comparability, this may limit the generalizability of findings to all PsA populations. Additionally, several other limitations should be acknowledged to enhance transparency. No neuroimaging techniques (e.g., MRI or fMRI) were employed to examine structural or functional brain changes associated with executive function impairments. As a result, we were unable to directly link cognitive performance to possible CNS alterations. Fatigue, mood disturbances (e.g., depression, anxiety), and sleep quality were not systematically assessed. These factors are known to affect executive functioning and may have acted as potential confounders. Finally, participants were recruited exclusively from an outpatient rheumatology clinic, which may introduce selection bias by favoring individuals with relatively better functional status and healthcare access. Therefore, the generalizability of our findings to broader or more severe patient populations may be limited. Additionally, the potential cognitive effects of DMARDs cannot be excluded, although both groups were comparable in terms of treatment exposure. Moreover, as this was a cross-sectional study, causality between disease characteristics and executive function outcomes cannot be established.

## 5. Conclusions

This study highlights that executive function impairments are more prominent in patients with RA than in those with PsA, despite both groups experiencing functional limitations and reduced quality of life. RA patients demonstrated significantly worse performance on neuropsychological tests assessing cognitive flexibility and inhibition (WCST, Stroop-TBAG, ADEXI), with these impairments closely associated with higher disease activity, older age, and longer disease duration. In contrast, PsA patients demonstrated relatively preserved executive functioning across disease activity levels, suggesting a potentially different neuroinflammatory impact compared to RA. While both conditions negatively affected daily functioning and quality of life, the broader cognitive burden observed in RA points to the need for more integrative disease management strategies. These findings emphasize the clinical value of incorporating routine cognitive and functional assessments into the care of RA patients, particularly those with moderate or high disease activity. Future longitudinal studies are warranted to explore underlying mechanisms further and to evaluate the potential benefits of targeted interventions aimed at preserving cognitive health in inflammatory arthritis.

## Figures and Tables

**Table 1:** Sociodemographic and clinical characteristics of patients with RA and PsA.

Variable	RA (*n* = 70)	PsA (*n* = 70)	*p*-Value
**Gender**			0.065
Female	54 (77.1%)	44 (62.9%)	
Male	16 (22.9%)	26 (37.1%)	
**Occupation**			**0.011**
Employed	12 (17.1%)	27 (38.6%)	
Homemaker	47 (67.1%)	31 (44.3%)	
Retired	10 (14.3%)	8 (11.4%)	
Student	1 (1.5%)	4 (5.7%)	
**Marital Status**			0.309
Married	57 (81.4%)	52 (74.3%)	
Single	13 (18.6%)	18 (25.7%)	
**Comorbidity**			0.307
No	49 (70.0%)	42 (60.0%)	
Yes	21 (30.0%)	26 (37.1%)	
**Medication**			0.085
csDMARD	41 (58.6%)	35 (50.0%)	
Biologic	5 (7.1%)	10 (14.3%)	
csDMARD + Biologic	22 (31.4%)	16 (22.9%)	
None	1 (1.4%)	6 (8.6%)	
**Age (mean ± SD)**	52.4 ± 11.7	48.7 ± 12.0	0.066
**Disease duration (years)**	12.4 ± 8.2	8.7 ± 6.6	**0.006**
**Years of education**	9.2 ± 4.1	10.6 ± 4.8	0.090
**DAS28-CRP**	2.7 ± 0.9	2.4 ± 1.0	**0.036**
**Laboratory Parameters**			
Urea (mg/dL)	33.8 ± 46.7	29.0 ± 13.4	0.927
Creatinine (mg/dL)	0.7 ± 0.2	0.7 ± 0.2	0.211
AST (U/L)	18.0 ± 5.4	20.2 ± 11.3	0.663
ALT (U/L)	19.1 ± 10.4	22.6 ± 13.5	0.181
CRP (mg/L)	23.9 ± 116.5	6.3 ± 12.0	0.154
ESR (mm/h)	17.5 ± 15.1	14.3 ± 11.6	0.317
Leukocyte (×10^9^/L)	8.7 ± 2.9	8.3 ± 2.3	0.772
Hemoglobin (g/dL)	13.0 ± 1.5	13.2 ± 2.1	0.200
Platelet (×10^3^/µL)	305.3 ± 82.3	280.0 ± 76.6	**0.031**

RA = Rheumatoid arthritis; PsA = Psoriatic arthritis; csDMARD = Conventional synthetic disease-modifying antirheumatic drug; DAS28-CRP = Disease Activity Score using 28-joint count and C-reactive protein; SD = Standard deviation; AST = Aspartate aminotransferase; ALT = Alanine aminotransferase; ESR = Erythrocyte sedimentation rate; CRP = C-reactive protein. Bold values indicate statistically significant results (*p* < 0.05).

**Table 2:** Assessment results in patients with RA and PsA.

Assessment Tool	RA (Mean ± SD)	PsA (Mean ± SD)	*p*-Value	Mean Difference	95%CI
**Stroop Test**					
Color–word time (s)	38.0 ± 13.8	30.0 ± 11.8	**<0.001**	8.00	[3.69, 12.31]
Self-corrections	3.0 ± 2.7	1.6 ± 1.8	**0.002**	1.41	[0.64, 2.19]
Errors	0.7 ± 1.2	0.1 ± 0.4	**<0.001**	0.61	[0.31, 0.92]
**Wisconsin Card Sorting Test (WCST)**					
Total responses	121.4 ± 14.4	113.8 ± 20.5	**0.012**	7.60	[1.64, 13.55]
Total errors	60.5 ± 24.7	46.3 ± 25.7	**0.001**	14.17	[5.73, 22.61]
Total correct responses	61.0 ± 16.8	67.2 ± 14.8	**0.029**	−6.20	[−11.50, −0.90]
Completed categories	2.7 ± 2.2	4.1 ± 2.0	**<0.001**	1.40	[−2.11, −0.69]
Perseverative responses	43.0 ± 32.9	32.5 ± 27.6	**0.042**	10.51	[0.35, 20.67]
Perseverative errors	34.8 ± 24.3	26.5 ± 21.4	**0.031**	8.31	[0.64, 15.99]
Non-perseverative errors	25.6 ± 18.6	20.9 ± 16.9	0.131	4.68	[−1.27, 10.63]
Perseverative errors (%)	27.7 ± 18.5	21.8 ± 15.9	**0.049**	5.91	[0.13, 11.71]
Responses to complete 1st category	49.3 ± 49.8	26.5 ± 32.9	**0.028**	22.81	[8.69, 36.93]
Responses at the conceptual level	40.8 ± 24.3	53.2 ± 20.0	**0.003**	−12.34	[−19.81, −4.88]
Conceptual level responses (%)	35.6 ± 23.8	50.0 ± 23.2	**0.001**	−14.37	[−22.25, −6.50]
Failure to maintain set score	1.0 ± 1.9	0.8 ± 1.3	0.794	0.21	[−0.35, 0.78]
**Adult Executive Functioning Inventory**					
Working memory	23.1 ± 7.1	21.7 ± 7.0	0.166	1.41	[−0.96, 3.79]
Inhibition	13.9 ± 2.5	12.9 ± 3.0	**0.013**	1.01	[0.08, 1.96]
**WHOQOL-BREF**					
Physical health	20.4 ± 6.0	22.5 ± 5.8	**0.041**	−2.05	[−4.06, −0.06]
Psychological health	20.2 ± 4.2	21.4 ± 4.3	0.066	−1.28	[−2.72, 0,15]
Social relationships	10.6 ± 1.3	11.0 ± 1.4	0.156	−0.37	[−0.05, 0.10]
Environmental health	29.2 ± 3.6	30.3 ± 3.6	0.054	−1.05	[−2.28, 0,16]
**Health Assessment Questionnaire (HAQ)**	10.7 ± 10.2	8.2 ± 9.0	0.165	2.42	[−0.80, 5.66]

RA = Rheumatoid arthritis; PsA = Psoriatic arthritis; WCST = Wisconsin Card Sorting Test; ADEXI = Adult Executive Functioning Inventory; WHOQOL-BREF = World Health Organization Quality of Life Instrument—Short Form; HAQ = Health Assessment Questionnaire; SD = Standard deviation. Bold values indicate statistically significant results (*p* < 0.05).

**Table 3:** Correlations of assessment results with age, disease duration, and DAS28-CRP in RA and PsA patients.

Measure	RA						PsA					
	Age		Disease Duration		DAS28-CRP		Age		Disease Duration		DAS28-CRP	
**Stroop Test**	r	*p*	r	*p*	r	*p*	r	*p*	r	*p*	r	*p*
Color–word time	0.383	**0.001**	0.110	0.363	−0.008	0.948	0.501	**0.000**	0.347	**0.003**	0.219	0.068
Self-corrections	0.160	0.185	0.193	0.112	−0.153	0.211	0.090	0.468	0.105	0.398	0.241	**0.049**
Errors	0.132	0.280	−0.059	0.632	0.501	**0.000**	0.084	0.497	−0.084	0.498	0.017	0.888
**WCST**												
Total responses	0.420	**0.000**	0.084	0.491	0.004	0.974	0.341	**0.004**	0.046	0.713	0.101	0.417
Total errors	0.406	**0.000**	0.269	**0.026**	0.110	0.368	0.299	**0.012**	0.172	0.164	0.025	0.838
Total correct responses	−0.281	**0.018**	−0.286	**0.017**	−0.095	0.436	0.065	0.592	−0.242	**0.049**	0.065	0.602
Completed categories	−0.325	**0.006**	−0.118	0.336	−0.050	0.683	−0.289	**0.015**	−0.116	0.349	−0.066	0.597
Perseverative responses	0.490	**0.000**	0.274	**0.023**	−0.029	0.816	0.292	**0.014**	0.242	**0.048**	0.130	0.294
Perseverative errors	0.498	**0.000**	0.275	**0.022**	−0.023	0.855	0.269	**0.025**	0.200	0.104	0.118	0.342
Non-perseverative errors	−0.096	0.429	0.013	0.913	0.270	0.025	0.140	0.249	−0.061	0.623	−0.036	0.770
Perseverative errors (%)	0.491	**0.000**	0.291	**0.015**	−0.031	0.801	0.227	0.058	0.220	0.074	0.109	0.378
Responses to complete 1st category	0.194	0.108	0.117	0.338	0.173	0.155	0.212	0.079	0.090	0.468	−0.008	0.950
Conceptual level responses	−0.319	**0.007**	−0.237	**0.050**	−0.137	0.261	−0.105	0.387	−0.190	0.123	0.002	0.985
Conceptual level responses (%)	−0.394	**0.001**	−0.223	0.066	−0.139	0.253	−0.315	**0.008**	−0.147	0.235	−0.051	0.683
Failure to maintain set	−0.135	0.264	0.173	0.155	−0.019	0.877	0.209	0.083	−0.081	0.515	0.071	0.569
**ADEXI**												
Working memory	−0.016	0.894	0.164	0.177	0.239	**0.048**	0.125	0.304	0.179	0.148	0.189	0.126
Inhibition	−0.096	0.427	−0.156	0.199	0.275	**0.022**	−0.151	0.212	0.190	0.124	−0.048	0.702
**WHOQOL-BREF**												
Physical health	−0.223	0.064	−0.169	0.165	−0.497	**0.000**	−0.214	0.075	–0.157	0.203	−0.287	**0.018**
Psychological health	−0.044	0.716	−0.182	0.135	−0.392	**0.001**	−0.051	0.678	–0.006	0.958	−0.186	0.133
Social relationships	0.084	0.490	−0.161	0.187	−0.135	0.270	−0.085	0.084	–0.135	0.276	−0.074	0.554
Environmental health	−0.072	0.552	−0.243	**0.044**	−0.302	**0.012**	0.033	0.078	0.042	0.737	−0.211	0.086
**HAQ**	0.209	0.082	0.240	**0.047**	0.670	**0.000**	0.176	0.146	0.096	0.438	0.521	**0.000**

RA = Rheumatoid arthritis; PsA = Psoriatic arthritis; DAS28-CRP = Disease Activity Score using 28-joint count and C-reactive protein; WCST = Wisconsin Card Sorting Test; ADEXI = Adult Executive Functioning Inventory; WHOQOL-BREF = World Health Organization Quality of Life Instrument—Short Form; HAQ = Health Assessment Questionnaire; r = Pearson or Spearman correlation coefficient; *p* = *p*-value. Bold values indicate statistically significant results (*p* < 0.05).

**Table 4:** Comparison of assessment results across DAS28-CRP subgroups in RA patients.

Measure	Remission (*n* = 36)	Low Activity (*n* = 9)	Moderate Activity (*n* = 24)	*p*-Value
**Stroop Test**				
Color–word time	39.2 ± 14.6	35.0 ± 11.0	37.2 ± 14.0	0.814
Self-corrections	3.5 ± 2.6	3.1 ± 3.2	2.3 ± 2.6	0.184
Errors	0.7 ± 1.0	0.7 ± 1.3	0.7 ± 1.4	0.770
**WCST**				
Total responses	123.3 ± 13.6	115.7 ± 17.3	120.3 ± 14.8	0.155
Total errors	61.5 ± 24.1	57.5 ± 31.3	59.5 ± 24.3	0.967
Total correct responses	61.8 ± 18.3	59.1 ± 20.7	60.7 ± 13.5	0.967
Completed categories	2.4 ± 2.1	4.0 ± 2.2	2.7 ± 2.2	0.185
Perseverative responses	48.8 ± 36.8	41.5 ± 32.1	36.1 ± 26.0	0.477
Perseverative errors	39.1 ± 26.7	33.1 ± 24.8	30.0 ± 19.9	0.484
Non-perseverative errors	22.3 ± 18.6	24.2 ± 19.7	29.5 ± 17.0	0.171
Perseverative errors (%)	31.0 ± 20.5	26.9 ± 18.5	24.0 ± 15.1	0.501
Responses to complete 1st category	51.2 ± 52.6	32.1 ± 37.5	49.7 ± 48.5	0.829
Responses at the conceptual level	40.7 ± 24.8	47.3 ± 23.4	39.5 ± 24.7	0.711
Conceptual level responses (%)	34.5 ± 23.1	43.4 ± 24.6	35.1 ± 25.1	0.531
Failure to maintain set	1.1 ± 2.0	0.4 ± 0.8	1.0 ± 2.0	0.663
**ADEXI**				
Working memory	21.7 ± 6.0	25.3 ± 7.1	23.7 ± 7.7	0.382
Inhibition	13.0 ± 2.2	14.5 ± 2.2	14.9 ± 2.5	0.043 *
**WHOQOL-BREF**				
Physical health	22.6 ± 6.1	20.4 ± 4.2	17.0 ± 5.2	0.002 *
Psychological health	21.1 ± 4.4	21.4 ± 2.2	18.2 ± 4.1	0.020 *
Social relationships	10.7 ± 1.2	10.7 ± 0.8	10.4 ± 1.7	0.757
Environmental health	29.9 ± 3.2	28.7 ± 3.4	28.4 ± 4.1	0.172
**HAQ**	6.0 ± 8.1	13.2 ± 7.6	17.1 ± 10.6	0.000 *

RA = Rheumatoid arthritis; PsA = Psoriatic arthritis; DAS28-CRP = Disease Activity Score using 28-joint count and C-reactive protein; WCST = Wisconsin Card Sorting Test; ADEXI = Adult Executive Functioning Inventory; WHOQOL-BREF = World Health Organization Quality of Life Instrument—Short Form; HAQ = Health Assessment Questionnaire; SD = Standard deviation. * Post hoc analyses using Bonferroni correction revealed significant pairwise differences between remission and moderate activity groups in ADEXI inhibition, WHOQOL-BREF physical and psychological health domains, and HAQ scores. Detailed effect sizes and confidence intervals are provided in the main text. Bold values indicate statistically significant results (*p* < 0.05).

**Table 5:** Comparison of assessment results across DAS28-CRP subgroups in PsA patients.

Measure	Remission (*n* = 43)	Low Activity (*n* = 10)	Moderate Activity (*n* = 14)	*p*-Value
**Stroop Test**				
Color–word time	30.0 ± 12.4	31.5 ± 14.2	29.7 ± 9.6	0.809
Self-corrections	1.7 ± 1.9	1.4 ± 1.4	1.7 ± 1.7	0.970
Errors	0.9 ± 0.4	0.3 ± 0.9	0.0 ± 0.0	0.490
**WCST**				
Total responses	113.4 ± 20.3	114.6 ± 20.8	115.4 ± 20.4	0.931
Total errors	47.2 ± 26.2	44.4 ± 23.7	46.5 ± 27.4	0.946
Total correct responses	65.8 ± 15.4	70.2 ± 12.1	68.8 ± 16.4	0.692
Completed categories	4.0 ± 2.0	4.4 ± 1.8	4.0 ± 2.2	0.908
Perseverative responses	34.0 ± 32.2	29.4 ± 15.4	31.5 ± 21.0	0.745
Perseverative errors	27.4 ± 24.5	25.0 ± 13.8	26.0 ± 17.4	0.861
Non-perseverative errors	21.6 ± 18.1	19.4 ± 11.6	20.5 ± 17.9	0.994
Perseverative errors (%)	22.8 ± 18.3	20.3 ± 9.9	20.9 ± 12.7	0.874
Responses to complete 1st category	29.2 ± 37.4	17.0 ± 7.7	26.0 ± 32.8	0.869
Responses at the conceptual level	52.1 ± 20.8	57.8 ± 15.1	52.8 ± 23.0	0.791
Conceptual level responses (%)	49.2 ± 23.7	53.2 ± 20.3	48.7 ± 24.7	0.916
Failure to maintain set	0.8 ± 1.5	0.6 ± 0.9	0.7 ± 1.1	0.934
**ADEXI**				
Working memory	20.8 ± 6.2	23.3 ± 6.6	22.4 ± 12.5	0.619
Inhibition	12.8 ± 3.1	13.0 ± 3.4	9.0 ± 2.8	0.934
**WHOQOL-BREF**				
Physical health	23.2 ± 5.9	21.0 ± 6.6	21.2 ± 5.7	0.323
Psychological health	22.0 ± 4.3	19.5 ± 5.7	21.1 ± 3.0	0.312
Social relationships	11.0 ± 1.3	10.7 ± 1.8	11.1 ± 1.4	0.945
Environmental health	30.7 ± 3.6	28.5 ± 4.2	30.6 ± 2.9	0.239
**HAQ**	5.4 ± 7.5	9.7 ± 6.0	13.9 ± 10.5	0.001 *

RA = Rheumatoid arthritis; PsA = Psoriatic arthritis; DAS28-CRP = Disease Activity Score using 28-joint count and C-reactive protein; WCST = Wisconsin Card Sorting Test; ADEXI = Adult Executive Functioning Inventory; WHOQOL-BREF = World Health Organization Quality of Life Instrument—Short Form; HAQ = Health Assessment Questionnaire; SD = Standard deviation. * Post hoc analyses using Bonferroni correction revealed significant pairwise differences between remission and moderate activity groups in HAQ scores. Detailed effect size and confidence interval are provided in the main text. Bold values indicate statistically significant results (*p* < 0.05).

**Table 6:** Comparison of assessment results according to medication category in all patients.

Measure	csDMARD (*n* = 76)	Biologic DMARD (*n* = 15)	Biologic + csDMARD (*n* = 38)	*p*-Value
**Stroop Test**				
Color–word time	34.8 ± 13.5	31.7 ± 8.5	33.0 ± 13.7	0.661
Self-corrections	2.5 ± 2.5	2.0 ± 2.2	2.1 ± 2.2	0.881
Errors	0.4 ± 0.9	0.5 ± 1.4	0.3 ± 0.7	0.953
**WCST**				
Total responses	116.9 ± 19.5	118.8 ± 16.4	119.4 ± 15.9	0.555
Total errors	53.7 ± 27.2	51.3 ± 22.9	55.0 ± 25.4	0.717
Total correct responses	63.0 ± 15.9	67.4 ± 15.0	64.3 ± 17.2	0.739
Completed categories	3.3 ± 2.2	3.8 ± 1.9	3.2 ± 2.3	0.717
Perseverative responses	39.0 ± 31.7	32.2 ± 19.7	37.2 ± 30.4	0.961
Perseverative errors	31.4 ± 24.0	27.0 ± 16.3	30.3 ± 22.7	0.967
Non-perseverative errors	22.3 ± 18.3	24.2 ± 14.1	24.6 ± 17.2	0.328
Perseverative errors (%)	25.3 ± 18.1	22.1 ± 12.1	24.5 ± 17.2	0.990
Responses to complete 1st category	37.4 ± 43.1	27.4 ± 30.4	44.2 ± 48.4	0.798
Responses at the conceptual level	46.5 ± 22.5	53.3 ± 16.5	43.7 ± 25.6	0.693
Conceptual level responses (%)	43.0 ± 25.1	46.4 ± 17.3	38.9 ± 25.1	0.480
Failure to maintain set	0.8 ± 1.7	0.9 ± 1.2	0.8 ± 1.6	0.897
**ADEXI**				
Working memory	21.8 ± 6.4	21.4 ± 7.8	23.3 ± 7.6	0.468
Inhibition	13.5 ± 2.9	13.4 ± 2.2	13.3 ± 2.7	0.938
**WHOQOL-BREF**				
Physical health	22.2 ± 5.4	23.6 ± 6.6	18.7 ± 5.7	0.009 *
Psychological health	21.3 ± 4.1	22.8 ± 3.8	19.0 ± 4.0	0.014 *
Social relationships	11.0 ± 1.3	11.2 ± 0.9	10.2 ± 1.6	0.197
Environmental health	29.9 ± 3.4	31.4 ± 3.4	28.7 ± 3.8	0.173
**HAQ**	7.0 ± 6.7	8.1 ± 9.9	14.7 ± 12.3	0.016 *

csDMARD = Conventional synthetic disease-modifying antirheumatic drug; bDMARD = Biologic disease-modifying antirheumatic drug; WCST = Wisconsin Card Sorting Test; ADEXI = Adult Executive Functioning Inventory; WHOQOL-BREF = World Health Organization Quality of Life Instrument—Short Form; HAQ = Health Assessment Questionnaire; SD = Standard deviation. * Post hoc analyses using Bonferroni correction revealed significant pairwise differences in WHOQOL-BREF physical and psychological domain scores, as well as HAQ scores, between the combination therapy group and the csDMARD and/or biologic DMARD groups. Detailed effect sizes and confidence intervals are provided in the main text. Note: Seven patients who were not receiving any medication were excluded from the analysis. Bold values indicate statistically significant results (*p* < 0.05).

## Data Availability

The data presented in this study are available on request from the corresponding author.
